# Visualization of macrophage subsets in the development of the fetal human inner ear

**DOI:** 10.3389/fimmu.2022.965196

**Published:** 2022-09-09

**Authors:** Claudia Steinacher, Lejo Johnson Chacko, Wei Liu, Helge Rask-Andersen, Werner Bader, Jozsef Dudas, Consolato M. Sergi, Tamilvendhan Dhanaseelan, Nadjeda Moreno, Rudolf Glueckert, Romed Hoermann, Anneliese Schrott-Fischer

**Affiliations:** ^1^ Inner Ear Laboratory, Department of Otorhinolaryngology, Medical University of Innsbruck, Innsbruck, Austria; ^2^ Department of Surgical Sciences, Section of Otorhinolaryngology and Head and Neck Surgery, Uppsala University, Uppsala, Sweden; ^3^ Anatomic Pathology Division, Children’s Hospital of Eastern Ontario (CHEO), University of Ottawa, Ottawa, Canada; ^4^ Biosciences Institute, Faculty of Medical Sciences, Newcastle University, Newcastle upon Tyne, United Kingdom; ^5^ Developmental Biology and Cancer, University College London (UCL) Great Ormond Street Institute of Child Health, University College London, London, United Kingdom; ^6^ Department of Anatomy, Histology & Embryology, Division of Clinical & Functional Anatomy, Medical University of Innsbruck, Innsbruck, Austria

**Keywords:** fetal human inner ear, macrophages, development, IBA-1, CD45, CD68, CD163, CX3CL1

## Abstract

**Background:**

Human inner ear contains macrophages whose functional role in early development is yet unclear. Recent studies describe inner ear macrophages act as effector cells of the innate immune system and are often activated following acoustic trauma or exposure to ototoxic drugs. Few or limited literature describing the role of macrophages during inner ear development and organogenesis.

**Material and Methods:**

We performed a study combining immunohistochemistry and immunofluorescence using antibodies against IBA1, CX3CL1, CD168, CD68, CD45 and CollagenIV. Immune staining and quantification was performed on human embryonic inner ear sections from gestational week 09 to 17.

**Results:**

The study showed IBA1 and CD45 positive cells in the mesenchymal tissue at GW 09 to GW17. No IBA1 positive macrophages were detected in the sensory epithelium of the cochlea and vestibulum. Fractalkine (CX3CL1) signalling was initiated GW10 and parallel chemotactic attraction and migration of macrophages into the inner ear. Macrophages also migrated into the spiral ganglion, cochlear nerve, and peripheral nerve fibers and tissue-expressing CX3CL1. The mesenchymal tissue at all gestational weeks expressed CD163 and CD68.

**Conclusion:**

Expressions of markers for resident and non-resident macrophages (IBA1, CD45, CD68, and CD163) were identified in the human fetal inner ear. We speculate that these cells play a role for the development of human inner ear tissue including shaping of the gracile structures.

## Introduction

The human inner ear has long been regarded as an immune-privileged region lacking immune responses. Recent studies, however show that despite a blood-labyrinth barrier, the human inner ear, including the cochlea, is populated by macrophages belonging to the innate immune system (1–5). These macrophages have been shown to play a role in local cochlear immune response following acoustic trauma or ototoxicity (3, 6, 7). In the mouse model, after acoustic overstimulation it was known that monocytes are able switch to macrophage shapes (8). They can be categorized into tissue resident and infiltrated macrophages. Monocytes may be recruited from the blood vessels after inflammatory signals. These monocytes differentiate in the tissue to so-called infiltrated macrophages. Resident macrophages live their entire lifespan in the tissue and can mediate signals to recruit other immune cells after inflammation or injury (8–10). Increased macrophages and fibrosis were observed after CI surgery, suggesting a role in wound healing and restoration (3, 11). Macrophages seem to play a dualistic role being both protective but also causing destructive responses leading to cell damage. Their exact role in the inner ear, during development and derivation in humans remains somewhat enigmatic. Dong et al, 2018 showed that in C57BL/6J mice the macrophages undergo a dynamic rearrangement with a decreasing in their number during postnatal development of the inner ear (12).

The aim of the present investigation was to analyse the distribution of macrophages and monocytes in the human inner ear during the development. It may increase our understanding of the roles during the complex process of cellular differentiation and maturation of the sensory organs.

For this purpose, we used different markers (IBA1, CD45, CD163 and CD68) to analyse the infiltration of macrophages and monocytes in the human inner ear during maturation and morphogenesis (13). IBA1, an ionized binding adaptor molecule1, is a specialized calcium binding protein that binds specific to the surface of microglia and macrophages. IBA1 is involved in resident macrophage phagocytosis and aids in membrane motility. This cytoplasmic marker occurs in podosomes and small multicellular complexes anchored to the extracellular matrix (2, 14, 15). The CD68 is a low-density lipoprotein (LDL) - binding protein and a lysosomal protein marker. This protein binds to the membrane of lysosomal cells like, macrophages, microglia and other mononuclear phagocytes. On the cell membrane  CD68 act as a scavenger protein for oxidizing LDL protein and is a component in the antigen-presenting system of immune cells (2). The CD163 is a specific marker for monocytes, macrophages and microglia cell of the monocyte lineage. CD163 serves as a transmembrane protein and acts as an endocytic receptor for the hemoglobin-haptoglobin complex. It also aids in cytokine assembly as a reaction to bacterial infection (2, 16). CD68 and CD163 can be used in combination with transcription factors like pSTAT1 (17), RBP-J (18) and CMAF (16) to discriminate and identify M1 (pro-inflammatory) and M2 (anti-inflammatory) macrophage polarization *in vivo* (16, 17, 19–21). The CD45 or leukocyte common antigen marker is a highly conserved receptor tyrosine phosphatase. This protein is expressed on leucocytes and hematopoietic cells like macrophages and dendritic cells. The protein is essentially involved in the modulation and regulation of immune cells, like T-cell activation/interaction, lymphocyte development or macrophage regulation (13, 22–24). In the adult inner ear CD45 immune reactive mononuclear phagocytes migrate towards cochlear regions following acoustic trauma (25).

We also analysed the expression of the chemokine Fractalkine in the inner ear at different time points. It is a protein encoded by the CX3CL gene. The membrane bound glycoprotein serves as a chemokine signal for neuron cells, microglia, circulating monocytes, dendritic cells and macrophages. Moreover, it serves as a chemoattractant to promote the migration of immune cells to injured tissue (4, 7, 9). Fractalkine signalling regulates macrophage activation in adult human cochlea enabling ganglion neuron and ribbon synapses survival following acoustic injury (4). The present study may give us more information about the mechanisms behind and cellular interplay during macrophage invasion and activity.

## Materials and methods

### Ethical approval of fetal specimens

Specimens aged between GW09 and GW12 used in this study were obtained immediately after legal abortion procedures according to the Austrian law (§97StGB of the Austrian Criminal Law as promulgated on 13^th^ November 1998, Federal Law Gazette I). Cadaver donations to the Division of Clinical and Functional Anatomy of the Medical University of Innsbruck for scientific and educational purposes occur only with the donor’s informed consent collected before death. The donors declare during their lifetime that their dead bodies are to be consigned to the anatomical institute for research purposes and the education and advanced training of medical doctors. All embryological body and tissue donations (between the gestational ages 9 to 12) are released to the anatomical institute by the legally entitled person (mother) accompanied by written consent.

In Austria, there is no requirement for a consent of the parents or relatives for a clinical autopsy performed in a medical institution § 25 KAKuG Leichenöffnung (Obduktion) (Krankenanstalten- und Kuranstaltengesetz) (26–28).

Additional specimens (between the gestational weeks 9 and 17) were provided by the UCL London and Newcastle branches of the HDBR: Joint MRC/Wellcome Trust (grant# MR/R006237/1) Human Developmental Biology Resource (http://hdbr.org). Fetal and embryonic tissue was collected, with informed consent, and distributed to research projects under ethical approval 18/NE/0290 from the North East-Newcastle & North Tyneside 1 Research Ethics committee for HDBR Newcastle, and 18/LO/022 from the Fulham Research Ethics Committee for HDBR UCL London. Specimens were certified by embryologists to exhibit no visible malformations and their embryological ages were differentiated by quantifying characteristics like crown-rump length, external and internal morphology and the estimated gynaecological age. All specimens were devoid of any external or internal congenital defects.

### Tissue preparation, histology and immunohistochemistry on paraffin sections

Twenty-Six human embryos and foetuses (GW09 x2, GW10 x3, GW11x2, GW12x3, GW13 x3, GW14 x2, GW15 x2, GW16 x3, GW17 x3, GW18 x3 and GW19 x1 as biological/technical replicates were done *via* sectioning) were used for this study. Tissue preparation, immunohistochemistry and fluorescence-based immunohistochemistry procedure on human cochlear section was described in previous publications (29–32).

Positive controls (e.g., small intestine, brain, and pancreas) were supplemented to each experiment. Negative controls are acquired by alternating the primary antibodies with reaction buffer or substituting them with isotype-matching immunoglobulins. Immunohistochemistry sections were digitally examined using a Zeiss AxioVision 4.1 microscope software coupled to an AxioCam HRc colour camera and an AxioPlan2 microscope (Zeiss, Jena, Germany). The fluorescence immunohistochemistry stained sections were digitalized at 40x and 63x magnifications using a TissueFAXS Plus System coupled onto a Zeiss^®^ Axio Imager Z2 microscope (Jena, Germany). Image acquisition was performed using the TissueFAXS software (TissueGnostics^®^, Vienna, Austria). The quantitative analysis of the amount and distribution of macrophages/monocytes was performed in GraphPad prism 9.

### Antibodies

Primary and secondary antibodies used for immunohistochemistry and fluorescence immunohistochemistry were listed in **Table 1**.

**Table 1:** The hosts, dilutions, and sources of antibodies utilized in the study.

Antibody	Type	Host	Dilution	Producer, Catalog Number
IBA-1	Monoclonal	Rabbit	1:400	Abcam. Ab178847
Anti-CD45 (LCA)	Monoclonal	Mouse	pre-diluted dispenser drop	Ventana Roche, Cat. 760-2505
CD68	Monoclonal	Mouse	1:50	Novus, NB-100-683
CD163	Monoclonal	Rabbit	1:500	Abcam, Ab182422
CollagenIV	Polyclonal	Goat	1:10	EMB Millipore, AB769
CX3CL1	Monoclonal	Mouse	1:100	RD System, MAB3651
TUBB3 (β-3 Tubulin)	Monoclonal	Mouse	8mg/µl	RD System, MAB1195
Alexa Fluoro Donkey Anti Rabbit 488	Polyclonal	Rabbit	1:200	Invitrogen, A21206
Alexa Fluoro Donkey Anti Rabbit 546	Polyclonal	Rabbit	1:200	Invitrogen, A21207
Alexa Fluoro Donkey Anti Mouse 488	Polyclonal	Mouse	1:200	Invitrogen, A21202
Alexa Fluoro Donkey Anti Mouse 546	Polyclonal	Mouse	1:200	Invitrogen, A10036
Alexa Fluoro Donkey Anti Goat 546	Polyclonal	Goat	1:200	Invitrogen, A11056

## Results

### IBA1 cells in the development of the human inner ear

**Figure 1:**
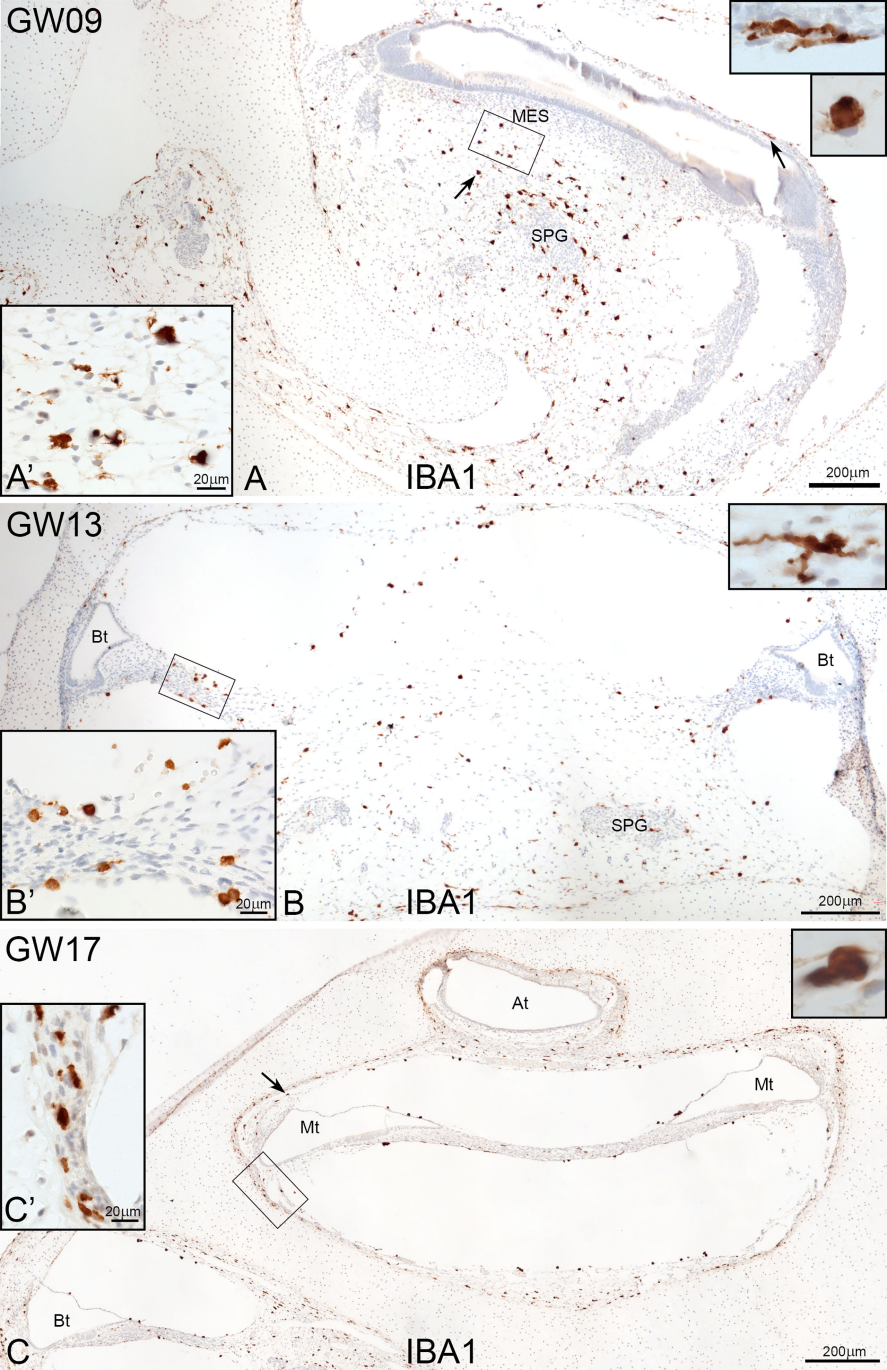
Overview of IBA1 positive macrophages during the development of human inner ear. **(A)** Macrophages are present around the spiral ganglion and the A’: mesenchymal tissue at GW09. **(B)** at GW13 (C0 and at GW17. Macrophages can see along the bony structure around the inner ear (Arrow and C’). Observations of amoeboid and transitional forms of IBA1 positive cells. (Inlets of **A–C** upper right).

IBA1 positive macrophages were present in the mesenchyme and connective tissue of human embryonic inner ear between GW09 to GW17 (**Figure 1**). Macrophage density did not significantly differ between early GW9 to late GW17 in the cochlea. Towards GW17, we observed several macrophages surrounding the inner ear’s otic capsule, which confirm the future entry of infiltrated macrophages into the sensory epithelium *via* the *scala vestibuli*, lateral wall and basilar membrane (**Figure 1C**).

**Figure 2:**
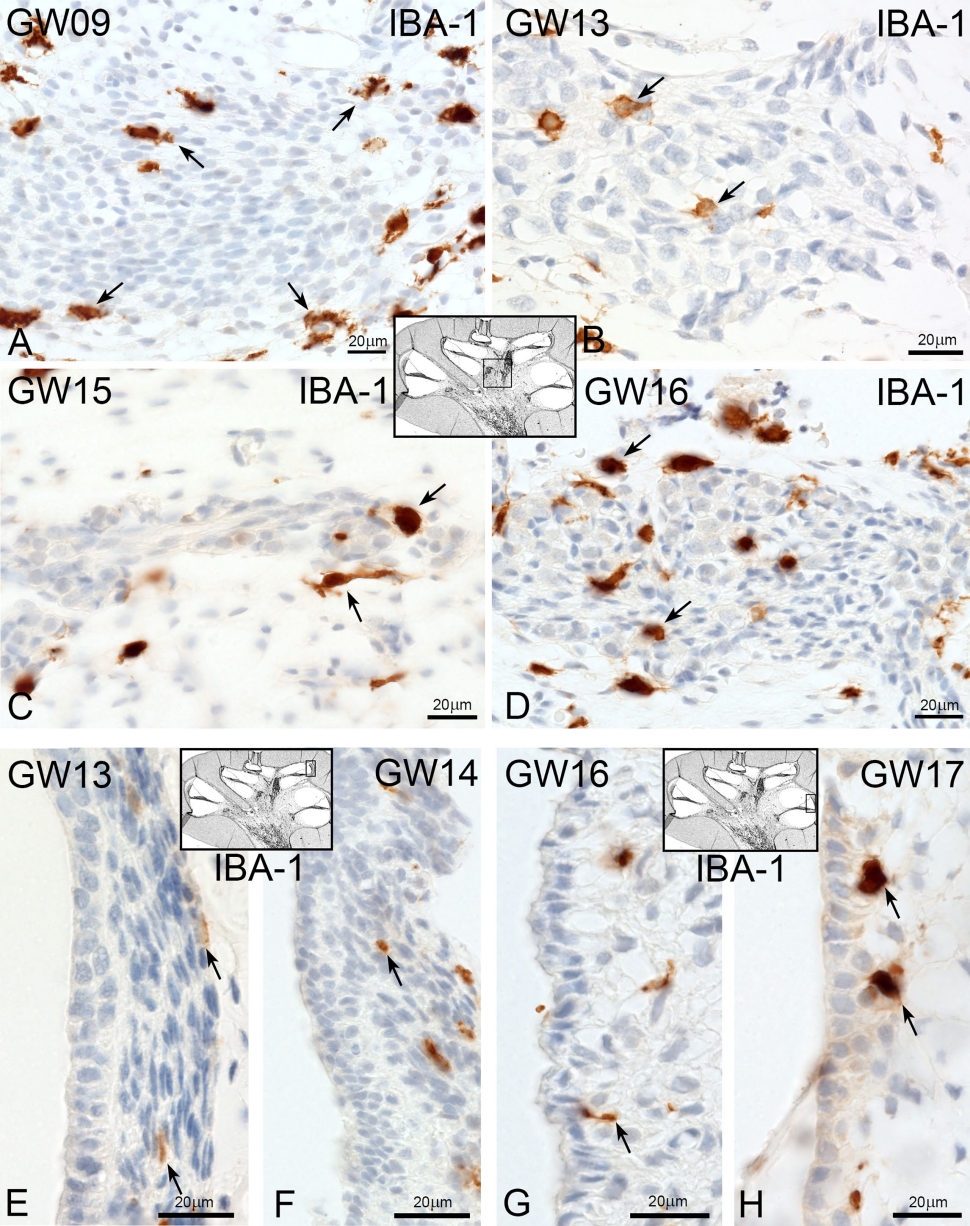
Macrophages at spiral ganglion and stria vascularis. **(A)** At early gestational weeks, the macrophages are present around the spiral ganglion tissue. **(B)** At GW13 macrophages start to infiltrate the spiral ganglion tissue and are present in and around at later gestational weeks (GW). **(C, D)**. **(E)** Macrophages pre-existing in the spiral ligament of the stria vascularis at GW13 and **(F)** at GW14. **(G)** First direct contact of macrophages with the stria vascularis (arrow). **(H)** and accumulation at later stages.

**Figure 3:**
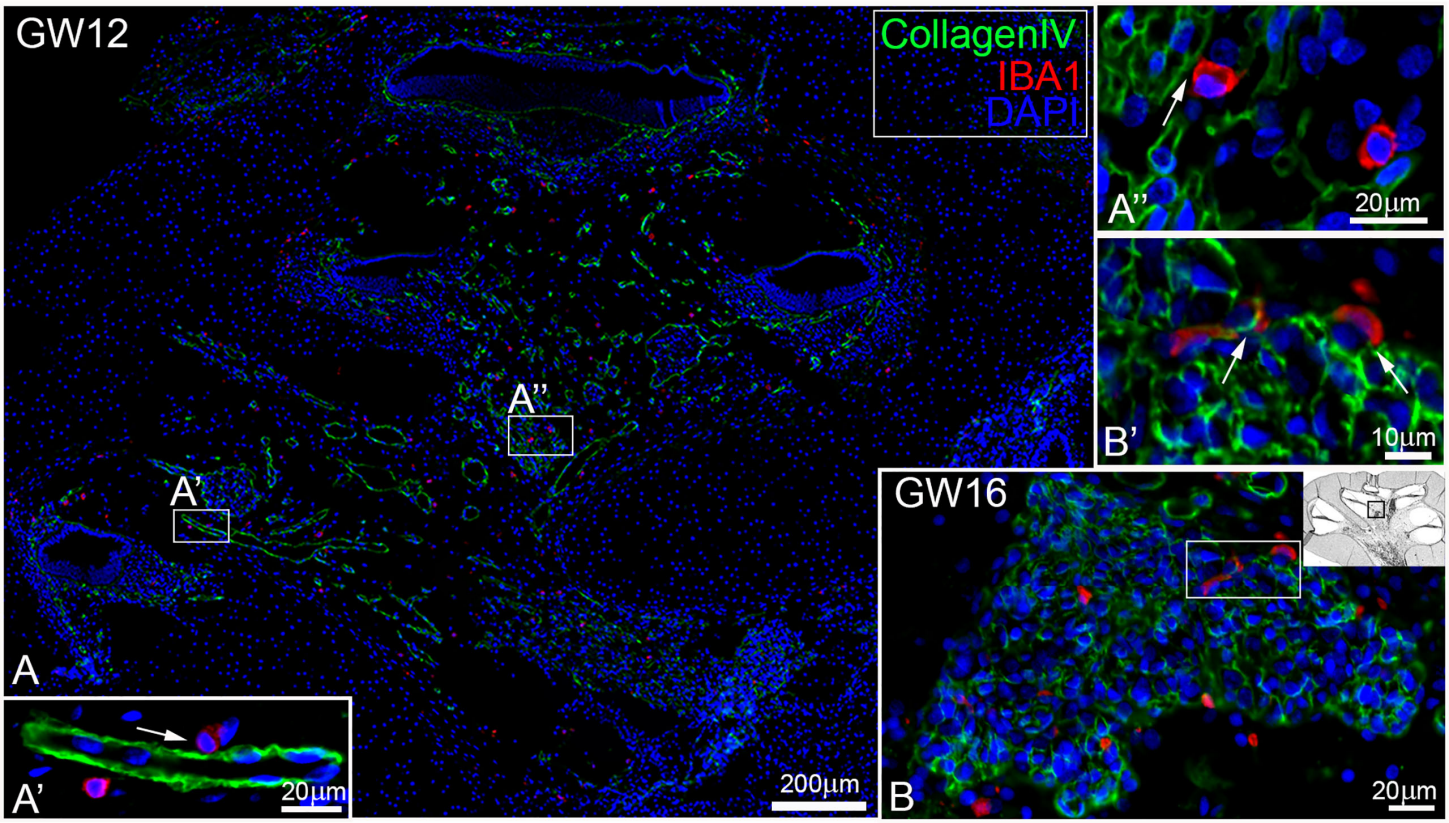
Macrophages are existing near blood vessel and nerve tissue. **(A)** Macrophages (red) are located A’’ near or A’ adjacent to blood vessel (green, arrow) and **(B)** in the spiral ganglion that were B’ attached to CollagenIV rich regions.

At GW09, macrophages with IBA1 positive staining were observed around nerve fibre tissues and spiral ganglions (**Figure 2A** arrows). By GW13, we saw the first migration into the spiral ganglia (**Figure 2B**). In later gestational weeks, an aggregation of ramiform and spheroid IBA1 positive macrophages was observed in and around the nerve tissue (**Figures 2C, D**). Before GW13, IBA1 positive macrophages were restricted to the mesenchyme of the future *stria vascularis*. After GW13, we observed the first steps of macrophage migration from mesenchyme into the *stria vascularis* epithelium (**Figures 2E–G** arrows). This migration of macrophages continued until GW16 (**Figure 2H**) when the first homing of IBA1 positive macrophage within the *stria vascularis* was observed. A direct infiltration of macrophages in the organ of Corti was not observed in all investigated developmental stages. Collagen IV and IBA1 co-staining demonstrated that macrophages were often found near extra cellular matrix (ECM) regions, blood vessels (**Figure 3A**) or nerve tissue (**Figure 3B**). We were able to localize IBA1 positive cells adjacent to the blood vessels (**Figure 3A’** and **3A’’** arrows) and nerve cells (**Figure 3B’**).

**Figure 4:**
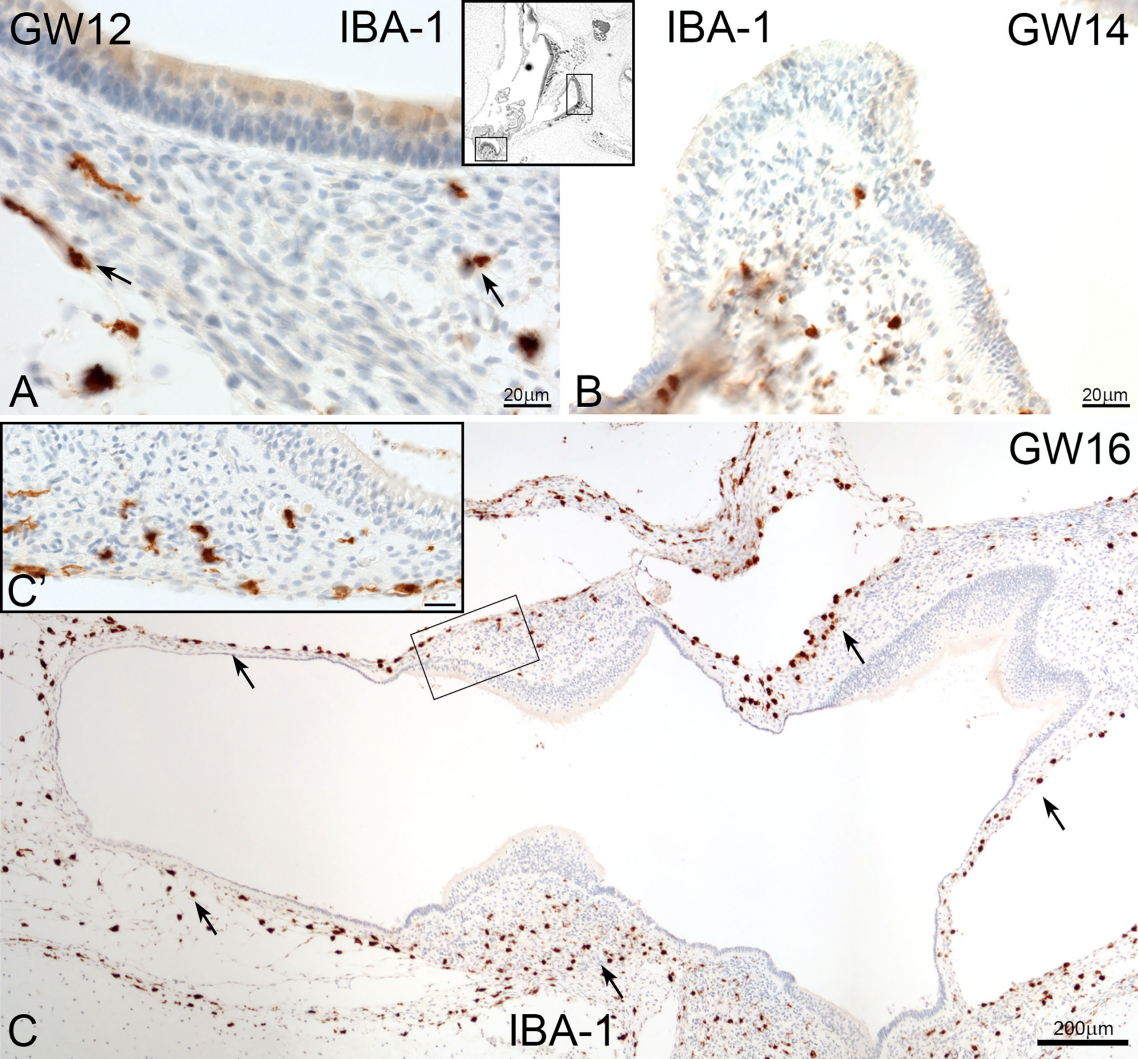
Macrophages in human foetus vestibulum. **(A)** IBA1 positive macrophages are present in the mesenchyme of the saccule, utricle and **(B)** crista ampullaris. (**C** and C’) High density of macrophages are found in the mesenchymal tissue of several regions of vestibulum.

In the vestibulum, the localization of IBA1 positive macrophages was restricted to the connective tissue (**Figure 4**). There was not seen a direct interaction with the vestibular sensory epithelium. Ramiform and spheroid macrophages accumulated only in mesenchymal tissue of utricle, saccule (**Figures 4A**) and the ampulla (**Figure 4B, C** arrows).

### Fractalkine ligand (CX3CL1) expression during morphogenesis

**Figure 5:**
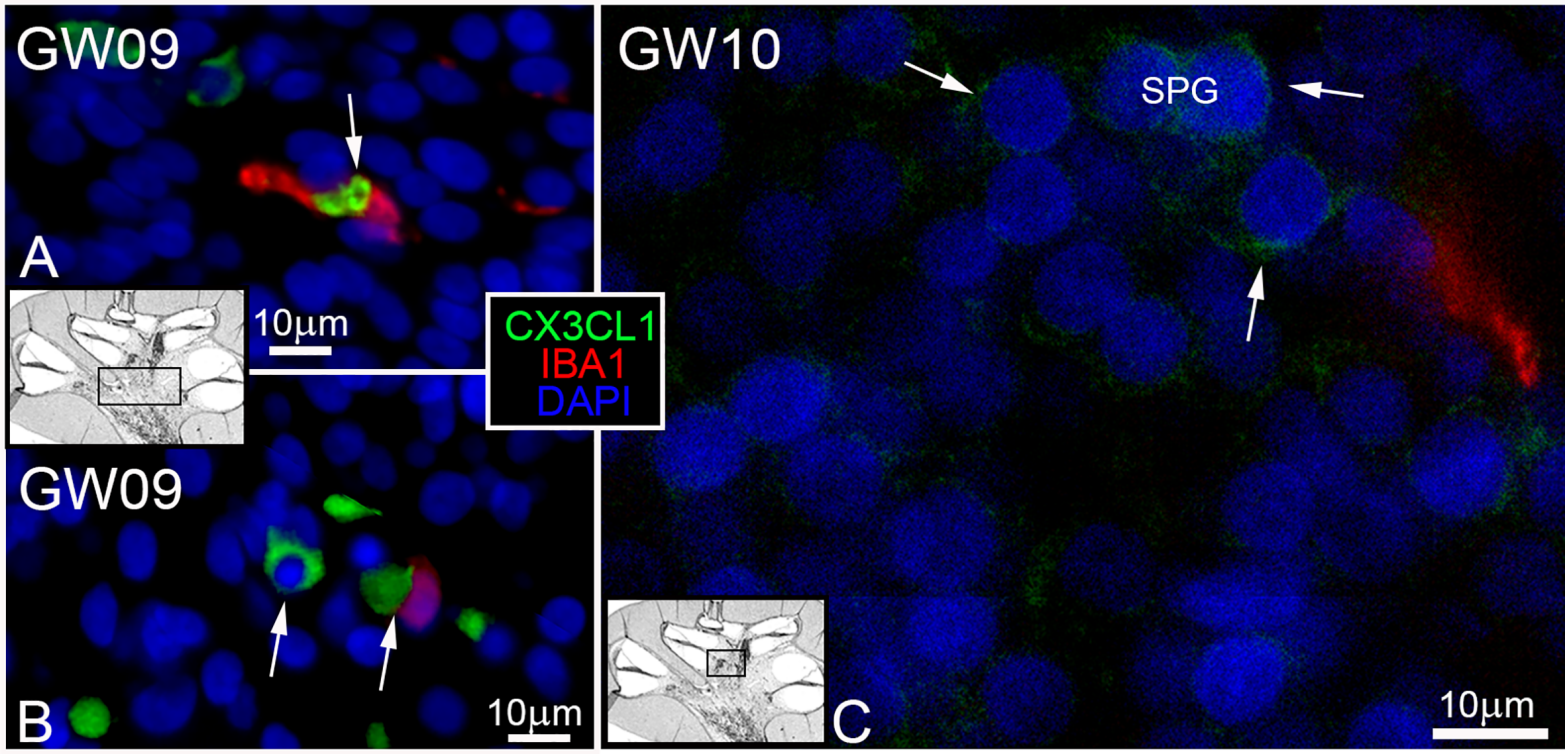
CX3CL1 ligand and IBA positive macrophages. **(A, B)** IBA1 macrophages (red) found in contact and near to CX3CL1 (green) positive cells in the mesenchyme. **(C)** First and diffuse staining (arrows) of Fraktalkine (CX3CL1) in spiral ganglion at GW10.

Surprisingly, the first appearance of the Fractalkine ligand (CX3CL1) was found at GW9. Cells positive for CX3CL1 were detected in the same timeframe as IBA1 positive macrophages in the mesenchymal tissue. IBA1 positive macrophages were in direct or near contact to CX3CL1 positive cells (**Figures 5A–C**, arrows). Cells within the spiral ganglion showed no expression of CX3CL1 at GW9. The first weak and diffuse distribution of the CX3CL11 in spiral ganglion cells was seen at GW10 (**Figure 5C** arrows). At later gestational weeks (GW12 to GW17), we detected an increase of the staining signal for CX3CL1 in neuronal cells, especially in spiral ganglion and cochlea nerve (data not shown). An interaction of CX3CL1 positive cells with IBA1 positive cells were also noted in the mesenchymal tissue and around nerve tissue of the inner ear in later gestational stages. Cells within the organ of Corti, *stria vascularis* and spiral ligament were devoid of immune reactivity of the Fractalkine marker.

### CD163 and CD68 positive cells during development of inner ear

**Figure 6:**
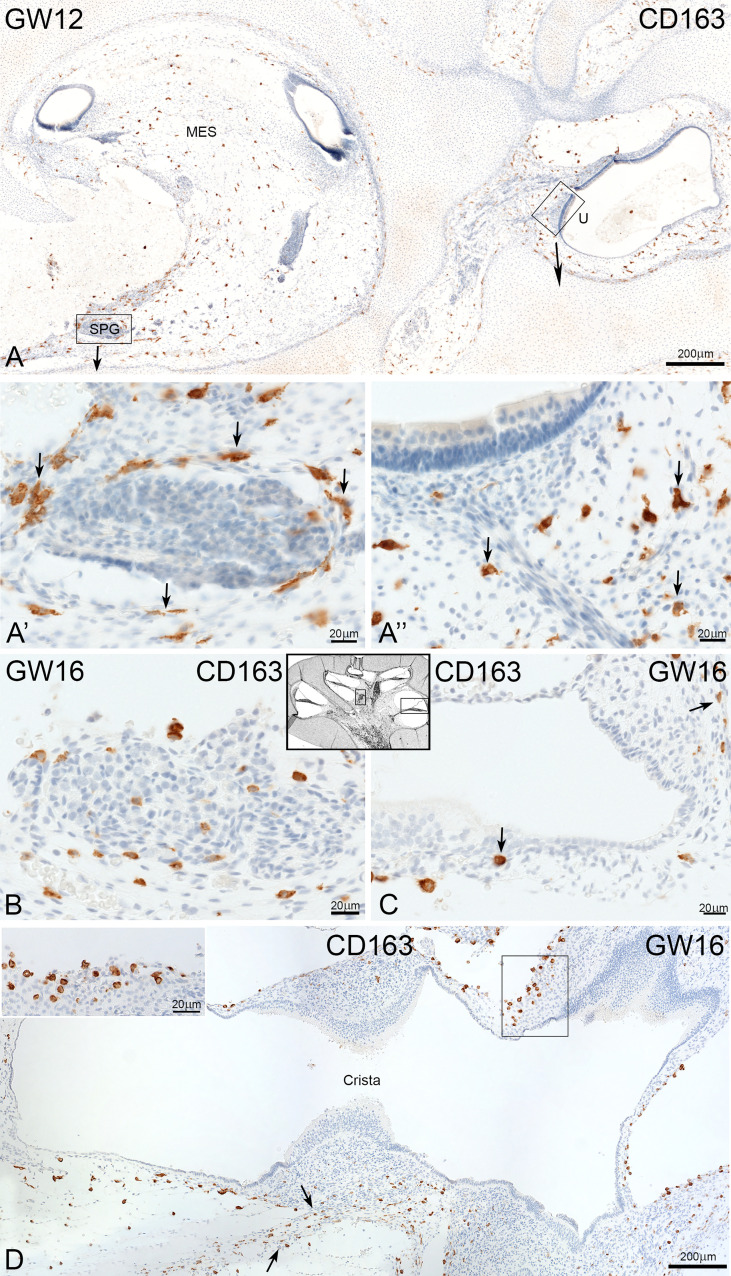
Expression of CD163 the development of human inner ear. **(A)** CD163 positive cells are found in the mesenchyme of cochlea and vestibulum at GW12. A’: Accumulation of CD163 cells around the spiral ganglion and A’’ under the sensor epithelium of utricle. **(B)** Infiltration of cells in the spiral ganglion **(C)** CD163 cells are present along the nerve fibre (arrow) and the spiral ligament of stria vascularis (arrow). **(D)** Staining show a moderate density of CD163 positive cells in the mesenchyme of vestibulum.

CD163 positive cells were present in the cochlear mesenchyme (**Figure 6A**) and vestibulum (**Figure 6D**). CD163 immuno-positive cells accumulated around the spiral ganglion at GW12 (**Figure 6A’**) and under the sensory epithelia of the utricle (**Figure 6A’’**). At GW16, we observed infiltration of CD163 positive cells in the spiral ganglion (**Figure 6B**). At the same time point, few CD163 positive macrophages were observed along the nerve fibre and spiral ligament of *stria vascularis* (**Figure 6C**, arrows).

**Figure 7:**
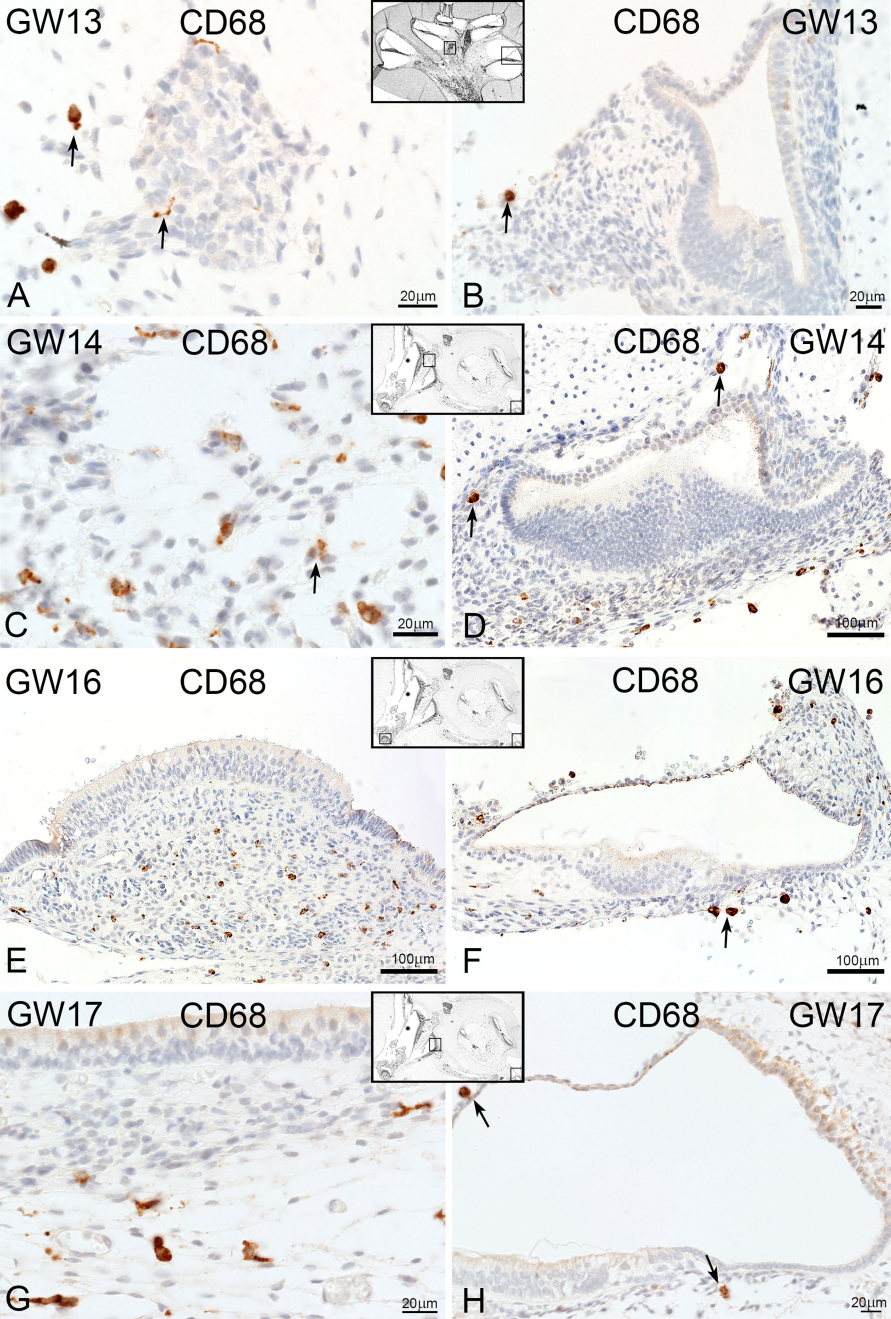
CD68 expression in developing human inner ear. **(A)** CD68 positive cells are localize around the spiral ganglion (arrows) at GW13 and **(B)** in the mesenchymal tissue of cochlea duct (arrow) and **(C)** vestibulum. **(D–H)** No change in expression and localization of CD68 positive cells at later weeks.

Macrosialin (CD68) positive macrophages/monocytes resided in the connective tissue of the cochlea and vestibulum (**Figure 7**). At GW13, CD68 stained cells surrounded and interacted with tissue-specific cells like neuronal cells of the spiral ganglion (**Figure 7A** arrow). Few CD68 positive cells were found near the cochlea ducts (GW13, **Figure 7B**, and arrow) and spiral ganglia (GW14, **Figure 7C**). Towards later gestational weeks (GW14, 16, and 17), few macrophages/monocyte cells were observed along the nerve fibers of the cochlear ducts (**Figure 7D**). At GW16, single CD68 positive cells were located at the basal membrane (**Figure 7F, H**). In the vestibulum, CD68 positive cells were found in the mesenchymal tissue of the ampulla (**Figure 7E**) and under the sensory epithelium of the utricle (**Figure 7G**) and saccule.

### CD45 in the foetus human inner ear

**Figure 8:**
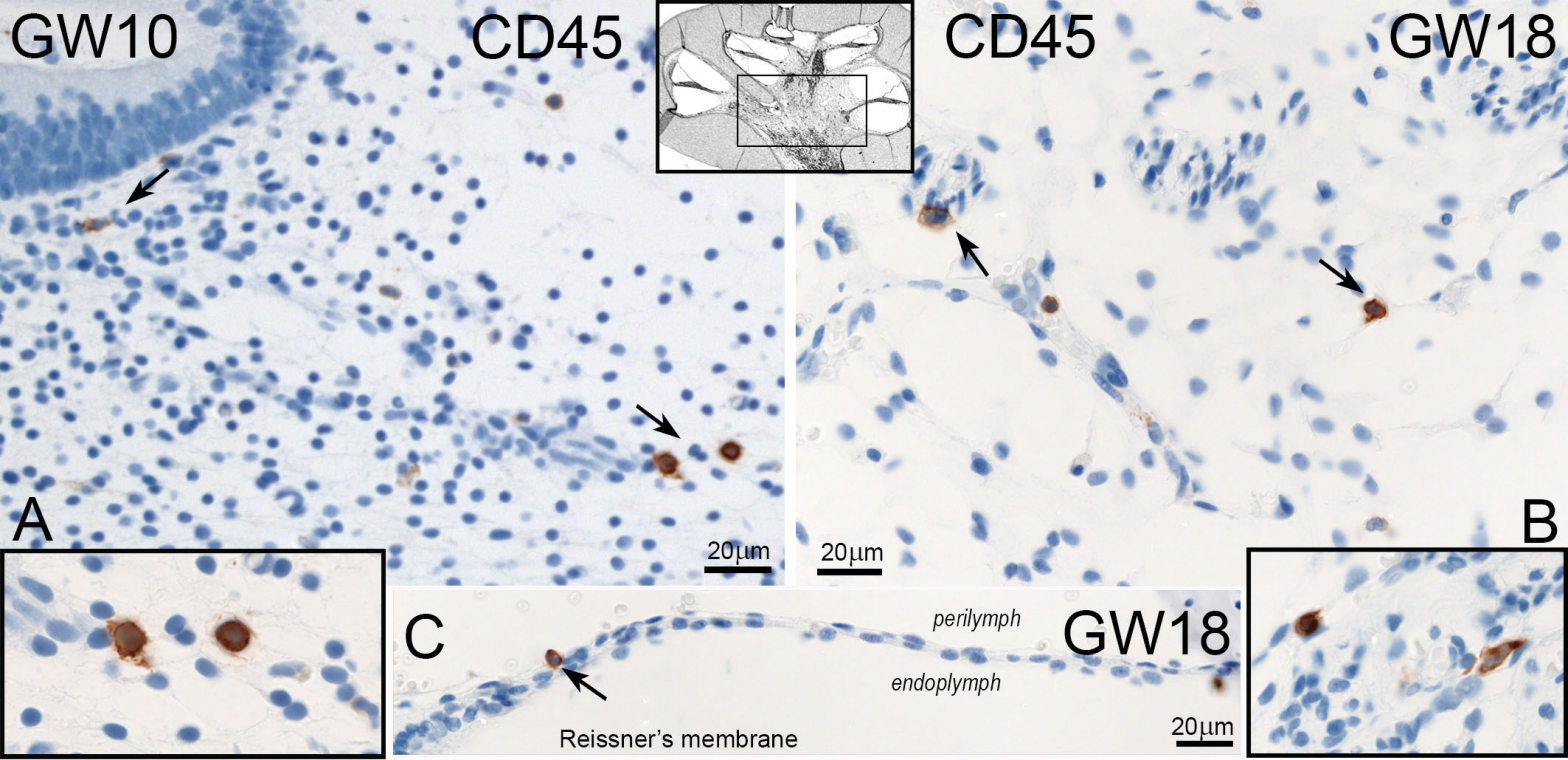
Expression of CD45 in the human inner ear. **(A)** CD45 positive cells are seen in the mesenchyme of vestibulum at GW10 with amoeboid forms (Inlet left). **(B)** Single cells of CD45 positive immune cells in the mesenchyme of the cochlea at GW18. **(C)** One CD45 stained cell is present in the Reissners’membrane at GW18.

**Figure 9:**
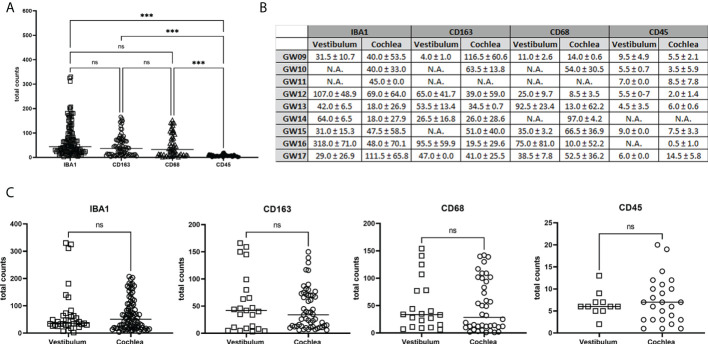
Quantification of total counts of IBA1, CD163, CD68 and CD45 positive immune cells in the development. **(A)** Comparison of total amounts of IBA1, CD163, CD68 and CD45. **(B)** Median and Standard deviation of counted cells for each developmental stages. **(C)** Vestibulum and Cochlea macrophages and monocytes distribution. Statistically significant is denoted by *, **, *** by representing P-value: *<0.05; **<0.01; ***<0.001. ns, not significant; N.A., not available.

The leukocyte common antigen protein CD45 was only present in the mesenchymal and connective tissue of cochlea and vestibulum (**Figure 8A, B**). We detected some cells near the spiral ganglion. During the investigated gestational weeks, we found just amoeboid forms (**Figure 8** inlet left and right). The distribution and number of CD45 positive immune cells were not significantly different in all developmental stages and between vestibulum and cochlea (**Figure 9B, C**). One non-mesenchymal CD45 cell was found at GW18 in the Reissner’s membrane (**Figure 8C**).

### Quantification of macrophages and monocytes in the development

The quantification results in **Figure 9A** showed a significance difference between the number of stained cells with CD45 related to IBA1, CD163 and CD68. Instead, between IBA, CD163 and CD68 were no statistical differences in the total counts. In detail of the total amount for each gestational week, we saw no different amount between vestibulum and cochlear region (**Figure 9B**). **Figure 9C** also showed no significant difference in the staining of IBA1, CD163, CD68 and CD45 between vestibulum and cochlea in the developing human inner ear.

## Discussion

To the best of our knowledge, this is the first systematic analysis of the distribution of macrophages at various developmental stages of the human inner ear (Overview **Table 2**). The first analyses using macrophage markers in the human adult inner ear was performed by O’Malley et al. in 2016 (2). Primary antibodies used against CD163, IBA1, CD45 and CD68 specific for macrophages/microglia to analyse paraffin-embedded human temporal bones. These cells were often closely associated with the neurons and at times, with the sensory cell areas of the auditory and vestibular epithelium (2, 3). Macrophage processes extend in different shapes throughout the labyrinth, including the endolymphatic duct and sac. Macrophages may be involved in phagocytosis of waste material in the endolymphatic duct and sac. They also aid in the digestion of luminal glycoproteins and immune reactions.

**Table 2:** Overview of summarized findings of immune cell distribution related to inner ear morphology and developmental stage in non-syndromic inner ear tissue.

Antibody	Overview
**IBA1**	• Between GW9 and GW17 Macrophages/Monocytes found in the mesenchyme and connective tissue with an accumulation around spiral ganglia • First infiltration at GW13 into spiral ganglion • Between GW9 and GW17 no direct infiltration of macrophages and monocytes into the Organ of Corti • Start of GW13 few macrophages observed in the *spiral ligament* near the fibrocytes with migration to *stria vascularis* region • In vestibulum macrophages and monocytes found in mesenchyme and connective tissue
**CD163**	• Mainly in the mesenchymal and connective tissue of cochlea and vestibulum
**CD68**	• Distinct macrophage population during all the stages examined in the mesenchyme and connective tissue of cochlea and vestibulum • At GW16 single cells are present at the basilar membrane
**CD45**	• Single cells with CD45 expression in the mesenchyme of cochlea and vestibulum. • At GW18 one CD45 positive cell in the Reissner’s membrane
**CX3CL1**	• At GW9 expression in the mesenchymal tissue of cochlea • At GW10 diffuse and weak expression in the spiral ganglion tissue

Macrophages are part of the innate immune system but can also initiate specific immune reactions harmful to the vulnerable inner ear. Therefore, the cells can be regarded as both saviours and foes depending on their activation mode (5). The human cochlea was recently shown to be endowed with immune cells such as CD4 and CD8 cells (33).

Macrophages may serve several functions and can even be found in the organ of Corti after acoustic damage. Macrophage-like perivascular resident melanocytes have been described in the mouse *stria vascularis* that were believed to influence fluid homeostasis in the cochlea *via* the intrastrial blood/labyrinth barrier (34). Studies reported that dendritic cells may reside within the human cochlea where they are engaged in tissue healing and cell preservation, maintaining the integrity of the fluid compartments (4, 35).

The present study shows that the human inner ear receives macrophages at an early stage suggesting that they play a key role in organ formation and cell differentiation. This incursion was correlated with the expression of Fractalkine, which is the ligand for a receptor expressed on the cell surface of the macrophages. Noteworthy was the infiltration of IBA1 positive cells in the spiral ganglion. IBA1, a known ionized calcium-binding adaptor molecule 1, is involved in membrane ruffling in phagocytes (2). Super-resolution microscopy (SR-SIM) have disclosed remarkable variants of IBA1 cells associated with the spiral ganglion cells. They are endowed with slender processes, can extend “synapse-like” specializations, migrate and support the surveillance of damaged cells, and give possible neurotrophic supply. Results suggested that the human auditory nerve is under stimulation of the resident macrophage system. The non-myelinated nerve soma may alleviate it. During embryonic development, the IBA1 positive cells may serve additional purposes such as waste disposal, stimulation of nerve generation and maturation. Transitional and amoeboid forms of macrophages were present around the spiral ganglion in mesenchymal tissue at GW09 and GW13. This coincided with the expression of Fractalkine, a chemoattractant protein, expressed on neuronal cells for promoting immune cell migration (7, 9) that observed as early as GW9 in the spiral ganglion. The macrophages may play a particular role in the human lateral wall where they infiltrate the stria vascularis and surround the blood vessels. Here, they express MHCII a major histocompatibility complex normally found on professional antigen-presenting cells. These cells may form a protective layer around the vessels to arrest antigen complexes from entering the tissue but they may also influence the fluid homeostasis and regulate ion content in the cochlear fluids, especially during a critical development. We hypothesize that a sudden increase in resident macrophage population positive for IBA1 during development is due to the cochlear blood vessels serving as a delivery system for these macrophages for the otherwise closed adult inner ear (36). The present study showed the essential periods for the invasion of macrophages in the stria vascularis. At GW16, macrophages made first contact and at GW17, IBA1 positive macrophages reached the epithelial cells of the future *stria vascularis*. By using CollagenIV staining it was also possible to relate the macrophages to the basal lamina of the blood capillaries as well as in the ganglion cells surrounding satellite cells. Thereby it was possible to detect their close relationship verifying the previous reports in the mature human cochlea demonstrating the close relationship and the large number of thin projections contacting the cell bodies as well as individual axons (5, 15). There was also an early invasion of IBA1 cells after GW12 and 14 under the sensory epithelium of the saccule, utricle and crista in the vestibular organ. However, after GW16 there was a heavy invasion suggesting that, at this time point, these cells play a particular role in cellular development and maturation of the vestibular epithelia.

The CD68 lysosomal marker (37, 38) and the human homolog of mouse Macrosialin may play a role as the scavenger of oxidized LDL and is essential for the antigen-presenting system (2). At GW13 CD68 expressing cells localized in and around the spiral ganglion and mesenchymal tissue below the cochlear duct. CD68 staining is also present in the mesenchymal tissue of vestibulum. Same staining pattern of CD68 was seen until gestational week 16/17 in vestibulum and cochlea in GW14.

The scavenger receptor molecule CD163, marker of monocytes, macrophages, and microglia, linked to cytokine production and innate sensor for bacteria (2, 13). CD163 positive cells appeared in the cochlear duct and vestibular organ as early as GW12. Cells were seen around the spiral ganglion and under the sensor epithelium of the utricle. Infiltration of CD163 positive cells were seen in the spiral ganglion at GW16. CD163 cells were also seen along the nerve fibre and in the spiral ligament of stria vascularis at GW16 suggesting that the cells play a similar role for the cellular organ development.

The CD45 common leucocyte antigen marker may be involved in cochlear repair to help to remove the cellular debris with antigen-induced immune response and tissue regeneration after acoustic trauma (39, 40). These suggest that CD45 could help to recruit other immune cells to obtain the homeostasis during morphological changes in the inner ear.

### The role of macrophages in fetal development of the Human Inner Ear

The present study shows the early invasion of macrophages in the human inner ear during development. It has become clear that these cells act not only as scavenger cells during development but can also protect by eliminating foreign substances. This also includes immune surveillance, inborn immunity, tissue repair and homeostasis (14, 41, 42). Furthermore, macrophages can help to remove cellular debris during the development, like supernumerary glial cells of nerve tissue. An abnormal number of nerve cells can lead to a deformation in the auditory nerve system, resulting in hearing dysfunction, as reported in mouse model studies (1, 43). Evidently, macrophages are of different sources such as bone marrow and other embryonic precursors. Their possible role as inflammatory mediators in the development of various disorders is beginning to arise (42). Studies on mouse and human adult cochlea indicates that macrophages and microglia are widely excluded from healthy inner ear tissue and/or help to obtain the homeostasis with housing in the specialized regions (9). Therefore, we should look even further into these fascinating cells housed in the human inner ear.

## Data availability statement

The raw data supporting the conclusions of this article will be made available by the authors, without undue reservation.

## Ethics statement

Fetal and embryonic tissue was collected, with informed consent, and distributed to research projects under ethical approval 18/NE/0290 from the North East-Newcastle & North Tyneside 1 Research Ethics committee for HDBR Newcastle, and 18/LO/022 from the Fulham Research Ethics Committee for HDBR UCL London. The patients/participants provided their written informed consent to participate in this study.

## Author contributions

CS: Performed immunostaining, collected data, analysed data, wrote manuscript. LJC: Collected data, analysed data, manuscript revision, wrote manuscript. WL, HR-A: Manuscript revision and discussion. WB: Manuscript revision. CS, JD: Analysed the data and manuscript revision. RG: Manuscript revision. RH, TD and NM: Specimen collection and preparation. AS-F: Supervision, authors read, reviewed and approved the final manuscript. All authors contributed to the article and approved the submitted version.

## Funding

This research has been financially supported by the K-regio project “eVITA” (electrical Vestibular Implant Tirol Austria) sponsored by EFRE (Dieses Projekt wird aus Mitteln des Europäischen Fonds für regionale Entwicklung kofinanziert). The Austrian Science Fund (FWF Austria) projects I 4811 (Neurotrophins in developing human inner ear and in HNSCC) & project I 4147-B (Modelling electrical stimulation of the human cochlear nerve), also supported this research.

## Conflict of interest

The authors declare that the research was conducted in the absence of any commercial or financial relationships that could be construed as a potential conflict of interest.

## Publisher’s note

All claims expressed in this article are solely those of the authors and do not necessarily represent those of their affiliated organizations, or those of the publisher, the editors and the reviewers. Any product that may be evaluated in this article, or claim that may be made by its manufacturer, is not guaranteed or endorsed by the publisher.

## Abbreviations

CI, Cochlear Implant; CX3CL1, C-X3-C Motif Chemokine Ligand 1; CD, Cluster of Differentiation; ECM, Extra Cellular Matrix; GW, Gestational Week; IBA1, Ionized Calcium binding adaptor molecule 1; U, Utricle; Sac, Saccule; SPG, Spiral ganglion.
